# Comparison of Adverse Events Among Home- vs Facility-Administered Biologic Infusions, 2007-2017

**DOI:** 10.1001/jamanetworkopen.2021.10268

**Published:** 2021-06-03

**Authors:** Matthew C. Baker, Yingjie Weng, Robert Fairchild, Neera Ahuja, Nidhi Rohatgi

**Affiliations:** 1 Division of Immunology and Rheumatology, Department of Medicine, Stanford University, Stanford, California; 2 Quantitative Sciences Unit, Division of Biomedical Informatics Research, Department of Medicine, Stanford University, Stanford, California; 3 Division of Hospital Medicine, Department of Medicine, Stanford University, Stanford, California

## Abstract

**Importance:**

Infusion reactions occur in 7% to 20% of patients receiving biologics. Home infusions are convenient and incur lower costs but may be associated with more adverse events; the safety of receiving biologic infusions for immune-mediated diseases at home remains unclear.

**Objective:**

To assess whether patients receiving home biologic infusions have increased adverse events requiring emergency department (ED) or hospital admission compared with patients receiving facility infusions.

**Design, Setting, and Participants:**

This retrospective cohort study used administrative claims data from a large national insurer for adult patients who received biologic infusions for immune-mediated disease between January 2007 and December 2017. Patients with hematologic malignant neoplasms or bone marrow transplantation were excluded. Data were analyzed from August 2019 to October 2020.

**Main Outcomes and Measures:**

ED or hospital admission on the same or next day after administration of a biologic infusion at home vs at a facility; secondary outcomes included discontinuation of the biologic after an ED or hospital admission and postinfusion mortality.

**Results:**

Of a total of 57 220 patients (mean [SD] age, 50.1 [14.8] years; 512 314 [68.1%] women) who received 752 150 biologic infusions (34 078 home infusions [4.5%] to 3954 patients and 718 072 facility infusions [95.5%] to 54 770 patients), patients who received home infusions were younger (mean [SD] age, 43.2 [13.2] vs 51.3 [14.8] years), more likely to be men (14 031 [41.2%] vs 225 668 [31.4%]), and had a lower Charlson comorbidity score compared with patients who received facility infusions (mean [SD] score, 0.5 [1.0] vs 1.1 [1.3]). Home infusions were associated with 25% increased odds of ED or hospital admission on the same or next day after the infusion (odds ratio [OR], 1.25; 95% CI, 1.09-1.44; *P* = .002) and 28% increased odds of discontinuation of the biologic after the ED or hospital admission (OR, 1.28; 95% CI, 1.08-1.51; *P* = .005). There was no difference in postinfusion mortality between home or facility infusions. The rates of adverse events were highest with home infusions of tocilizumab (48 of 481 infusions [10.0%]), vedolizumab (150 of 2681 infusions [5.6%]), and infliximab (1085 of 20 653 infusions [5.3%]), although the number of tocilizumab and vedolizumab infusions was low.

**Conclusions and Relevance:**

In this study, biologic infusions administered at home, compared with those administered at a facility, were associated with increased adverse events requiring escalation of care. Because the number of home infusions has increased and is expected to continue to rise, the safety implications of administering biologic infusions at home needs to be further assessed.

## Key Points

**Question:**

Do patients who receive biologic infusions at home have more adverse events requiring escalation of care compared with patients who receive biologic infusions at a facility?

**Findings:**

In this cohort study of 57 220 patients with immune-mediated disease who received 752 150 biologic infusions, home infusions were associated with 25% increased odds of emergency department or hospital admission on the same or next day after infusion compared with facility infusions and 28% increased odds of permanent discontinuation of the biologic after emergency department or hospital admission.

**Meaning:**

These findings suggest that the safety implications of administering biologic infusions at home need to be assessed further.

## Teaser

This cohort study of patients who received biologic infusions for immune-mediated diseases examines the association of infusions received at home with admission to the hospital or emergency department within the next day.

## Introduction

Immune-mediated diseases affect less than 7% of the population but are responsible for a substantial proportion of health care utilization and spending.^1,2,3^ Biologics are highly effective treatments that target specific elements of the immune system to reduce immune cell activity and inflammation in patients with immune-mediated disease.^4^ While biologics generally have a good safety profile, acute infusion reactions within 24 hours of administration have been reported in 7% to 20% of patients.^5,6^ Infusion reactions most commonly occur after the first or second infusion of a given biologic.^6,7,8,9^ These acute infusion reactions range from mild (eg, headache, flushing, palpitations, nausea) to severe (eg, hypotension, stridor, anaphylaxis) and can be life-threatening.^8,10,11,12,13,14,15^

Increasingly, biologic infusions are being administered at home instead of at a facility.^16^ Biologics are expensive; they accounted for 38% of the total prescription drug spending in the US in 2015 and 70% of the growth in drug spending between 2010 and 2015.^3^ Infliximab, one of the most commonly prescribed biologics, costs approximately $6500 per facility-based infusion.^2^ Up to 16% of the costs associated with biologics can be attributed to facility overhead.^2^ Hence, there is an economic incentive to administer biologics at home instead of at a facility. In addition, home infusions are often more convenient for patients and caregivers.

Currently, because of sheltering-in-place and social distancing during the COVID-19 pandemic, a further increase in home infusions is expected.^17,18,19,20^ However, the safety implications of administering biologic infusions at home remain unclear. To our knowledge, no large-scale studies have been performed to evaluate the safety of home biologic infusions compared with facility biologic infusions in patients with immune-mediated diseases. In this study of adult patients with immune-mediated disease across the US, we assessed whether administration of biologic infusions at home instead of at a facility was associated with an increase in adverse events requiring escalation of care.

## Methods

This study adhered to the Strengthening the Reporting of Observational Studies in Epidemiology (STROBE) reporting guideline for cohort studies. The Stanford University institutional review board exempted this study from review and the requirement for informed consent because it used a deidentified database.

### Data Source

We used data from the Optum Clinformatics Data Mart, an administrative health claims database from a large national insurer, from January 1, 2007, to December 31, 2017. The data are derived from administrative health claims of more than 15 million individuals annually, including privately insured and Medicare Advantage Part D members. This database includes patients from states nationwide.

### Study Population

We included patients aged 18 years and older who had at least 1 *International Classification of Diseases, Ninth Revision (ICD-9)* or *ICD-10* code for an immune-mediated disease and at least 1 J-code (Healthcare Common Procedure Coding System codes that identify injectable drugs that cannot be self-administered) for a biologic infusion (eFigure 1 and eTable 1 in the Supplement). We excluded infusions given to patients with a history of hematologic malignant neoplasms or bone marrow transplantation and those who did not have place of service documented in our database (eTable 2 in the Supplement).

 We defined home and facility infusions as biologic infusions for which the place of service was documented as home and office, respectively. We excluded biologic infusions for which the place of service was documented as a hospital (<1% of infusions).

### Data Collection

For patients receiving home or facility infusions, we extracted data on age, sex, year of infusion, disease specialty, and name of the infusion agents. We used the Quan-Deyo method to calculate the Charlson comorbidity score for each patient.^21^ For additional risk adjustment, we collected data on the conditions that comprise the Segal frailty index.^22^

We also extracted data on the use of concomitant glucocorticoids using patient-level data to define infusion episodes. For the glucocorticoid analysis, an episode was defined as the period of time a patient received infusions of a given biologic. If the patient was switched to a different biologic or if 3 or more months elapsed between infusions of the same biologic (≥12 months for rituximab), the period of use that followed was designated as a new episode. For glucocorticoid use assessment, we excluded episodes in which the place of service switched between home and facility for a given biologic (1.3% of the episodes). All systemic glucocorticoids were converted to an equivalent dose of prednisone. We viewed concomitant glucocorticoid use as an important baseline characteristic, as we expect patients with less severe immune-mediated disease to have less glucocorticoid use.

### Primary Outcome

Our primary outcome was emergency department (ED) or hospital admission after the administration of a biologic infusion at home vs at a facility. We considered this escalation of care as an adverse event. In the Optum database, patients admitted to the hospital after being seen in the ED are counted as hospital admissions only. We included admissions on the same or next day after the infusion to capture the 24-hour duration when an acute infusion reaction could occur.

### Secondary Outcomes

Our secondary outcomes included (1) discontinuation of the biologic following an ED or hospital admission after an infusion (signaling a potentially direct association between the biologic infusion and the admission) and (2) postinfusion mortality. We did not have access to the date of death; thus, we included patients who died during the same month or the month following the infusion.

### Subgroup Analysis

We compared patients who had an ED or hospital admission after home or facility infusion with those who did not have an ED or hospital admission. Evaluated differences included demographic characteristics, clinical characteristics, disease specialty, and infusion agent.

### Statistical Analysis

Baseline characteristics for patients receiving biologic infusions at home and at a facility were described. Standardized mean differences (SMD) were calculated using the tableone package in R.

For our primary analysis of ED or hospital admission after the administration of a biologic at home vs at a facility, we used infusion-level data. Generalized estimating equations (GEE) were used to assess the association between the place of infusion and ED or hospital admission while adjusting for age, sex, Charlson comorbidity score, year of infusion, disease specialty, the frailty conditions included in the Segal frailty index,^22^ the top 7 most common infusion agents (comprising 96.6% of all the biologics prescribed in our database), and glucocorticoid use in the past 30 days. GEE was used to adjust the standard error for intrapatient correlations. We used binomial distribution and logit link and assumed an unstructured variance-covariance matrix. Assumptions made under exchangeable, autoregressive, and independent structure as sensitivity analyses yielded similar results. For our secondary analysis of the rate of biologic discontinuation following ED or hospital admission after an infusion and postinfusion mortality, fewer covariates were selected (age, sex, Charlson comorbidity score, year of infusion, and disease specialty) owing to the small number of events.

 Patients with missing data for age (17 patients) or sex (1 patient) were excluded from the analysis. Odds ratios (ORs), 95% CIs, and *P* values were reported from the regression models. All analyses were conducted using R version 3.6.1 (R Project for Statistical Computing) and geepack. Charlson comorbidity scores were calculated using the icd package implemented in R version 4.0.6.^21^ We applied Bonferroni corrections for multiple comparisons for our primary outcomes. Statistical significance was set at *P* < .0125, and all tests were 2-tailed.

## Results

### Study Cohort

**Figure 1.**
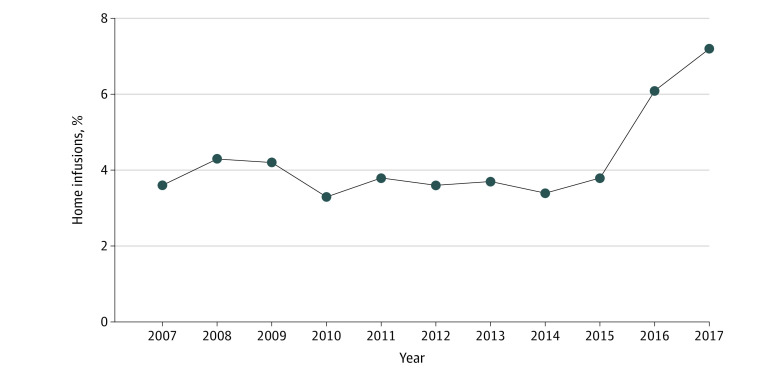
Annual Percentage of Infusions Delivered at Home, 2007 to 2017

Our cohort consisted of 57 220 patients with immune-mediated diseases (mean [SD] age, 50.1 [14.8] years; 512 314 [68.1%] women) who received 752 150 biologic infusions between 2007 and 2017 (eFigure 1 in the Supplement). A total of 34 078 infusions (4.5%) were administered at home and 718 072 infusions (95.5%) were administered at a facility (Table 1). Between 2015 and 2017, there was an increase in home infusions from 3.8% to 7.2% (Figure 1).

**Table 1.** Baseline Characteristics of Patients Receiving Biologic Infusions at Home or at a Facility

Characteristics	Infusions, No. (%)			SMD
	Total cohort	Home	Facility	
Infusions	752 150 (100)	34 078 (4.5)	718 072 (95.5)	NA
Patients	57 220 (100)	3954 (6.9)	54 770 (95.7)	NA
Age, mean (SD), y	50.1 (14.8)	43.2 (13.2)	51.3 (14.8)	0.583
Sex				0.205
Women	512 314 (68.1)	20 047 (58.8)	492 267 (68.6)	
Men	239 699 (31.9)	14 031 (41.2)	225 668 (31.4)	
Charlson comorbidity score				0.661
0	294 487 (39.2)	23 236 (68.2)	271 251 (37.8)	
1-2	368 079 (48.9)	9167 (26.9)	358 912 (50.0)	
3-4	60 882 (8.1)	828 (2.4)	60 054 (8.4)	
≥5	19 124 (2.5)	326 (1.0)	18 798 (2.6)	
Missing	9578 (1.3)	521 (1.5)	9057 (1.3)	
Charlson comorbidity score, mean (SD)	1.0 (1.3)	0.5 (1.0)	1.1 (1.3)	0.506
Glucocorticoid use in past 30 d	97 997 (13.0)	3016 (8.9)	94 981 (13.2)	0.140
Disease specialty				
Rheumatology	436 228 (58.0)	6965 (20.4)	429 263 (59.8)	0.876
Gastroenterology	191 076 (25.4)	19 782 (58.0)	171 294 (23.9)	0.742
Neurology	104 637 (13.9)	6834 (20.1)	97 803 (13.6)	0.173
Dermatology	18 120 (2.4)	722 (2.1)	17 398 (2.4)	0.020
Hematology/oncology	10 634 (1.4)	5 (<0.1)	10 629 (1.5)	0.171
Nephrology	280 (<0.1)	3 (<0.1)	277 (<0.1)	0.019
Infusion agent				1.499
Infliximab	394 638 (52.5)	20 653 (60.6)	373 985 (52.1)	
Abatacept	112 250 (14.9)	1879 (5.5)	110 371 (15.4)	
Natalizumab	102 390 (13.6)	6693 (19.6)	95 697 (13.3)	
Rituximab	41 130 (5.5)	400 (1.2)	40 730 (5.7)	
Tocilizumab	38 839 (5.2)	481 (1.4)	38 358 (5.3)	
Belimumab	22 021 (2.9)	513 (1.5)	21 508 (3.0)	
Vedolizumab	15 057 (2.0)	2681 (7.9)	12 376 (1.7)	
Golimumab	9697 (1.3)	43 (0.1)	9654 (1.3)	
Ustekinumab	4467 (0.6)	95 (0.3)	4372 (0.6)	
Pegloticase	1387 (0.2)	93 (0.3)	1294 (0.2)	
Canakinumab	154 (<0.1)	54 (0.2)	100 (<0.1)	
Unclassified biologic	10 108 (1.3)	486 (1.4)	9622 (1.3)	

Abbreviations: NA, not applicable; SMD, standardized mean difference.

Patients who received home infusions were younger (mean [SD] age, 43.2 [13.2] years vs 51.3 [14.8] years), more likely to be men (14 031 [41.2%] vs 225 668 [31.4%]), and had a lower mean (SD) Charlson comorbidity score (0.5 [1.0] vs 1.1 [1.3]) compared with patients who received facility infusions (Table 1). The top 3 biologics administered at home were infliximab (20 653 [60.6%]), natalizumab (6693 [19.6%]), and vedolizumab (2681 [7.9%]), and the top 3 biologics administered at a facility were infliximab (373 985 [52.1%]), abatacept (110 371 [15.4%]), and natalizumab (95 697 [13.3%]).

Compared with patients who received facility infusions, those who received home infusions had lower glucocorticoid use (988 [35.8%] vs 30 687 [46.5%]), but the median glucocorticoid dose was higher (median [interquartile range {IQR}] prednisone equivalent, 15.0 [7.0-25.0] mg/d vs 11.3 [6.7-21.0] mg/d) (Table 2). There was no difference in the number of days on glucocorticoid treatment between patients receiving infusions at a facility or at home.

**Table 2.** Concomitant Glucocorticoid Use During Home vs Facility Biologic Infusion Episodes^a^

	Infusions, No. (%)		
	Total cohort	Home	Facility
Episodes	68 763	2757	66 006
Patients	56 028	2623	53 561
Infusions per episode, mean (SD)	9.5 (12.5)	7.5 (9.0)	9.6 (12.6)
Patients with any glucocorticoid prescription, No. (%)	31 675 (46.1)	988 (35.8)	30 687 (46.5)
Glucocorticoid use, median (IQR), d	60 (30-120)	60 (30-120)	60 (30-120)
Glucocorticoid, median (IQR), mg/d	11.4 (6.7-21.2)	15.0 (7.0-25.0)	11.3 (6.7-21.0)

Abbreviation: IQR, interquartile range.

^a^ An episode was defined as the period of time receiving any given infusion; a new episode was determined if a patient started receiving another infusion 3 months or longer from the last infusion (≥12 months for rituximab). Glucocorticoids were converted to an equivalent dose of prednisone.

Of the 3954 patients who received home infusions, 1009 (25.5%) received their first infusion at a facility, followed by subsequent infusions of the same biologic at home (eFigure 2 in the Supplement). Of the 54 770 patients who received facility infusions, 54 275 (99.1%) received their first infusion at a facility.

### Primary Outcome

**Figure 2.**
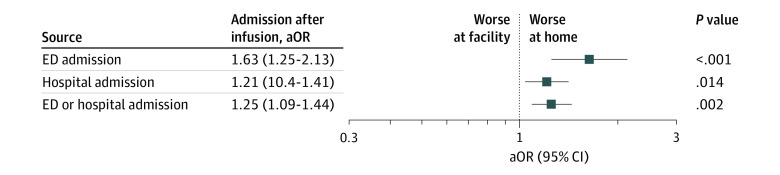
Association of Home vs Facility Biologic Infusion and ED or Hospital Admission on the Same or Next Day ED or hospital admissions included occurred the same day as or the day after a biologic infusion. aOR indicates adjusted odds ratio; ED, emergency department.

Of the 34 078 infusions that were administered at home, ED or hospital admission on the same or next day occurred after 1496 (4.4%) of the infusions (Table 3). Of the 718 072 infusions that were given at a facility, ED or hospital admission on the same or next day occurred after 25 048 (3.5%) of the infusions. After adjusting for age, sex, Charlson comorbidity score, year of infusion, disease specialty, Segal frailty index conditions, the top 7 most common infusion agents, and glucocorticoid use in the past 30 days, infusions administered at home were associated with 25% increased odds of ED or hospital admission on the same or next day after the infusion compared with infusions administered at a facility (OR, 1.25; 95% CI, 1.09-1.44; *P* = .002) (Table 3 and Figure 2). In terms of the absolute difference, 0.9 per 100 more individuals were admitted to the ED or hospital on the same or next day after infusions were administered at home compared with those administered at a facility.

**Table 3.** Association of Home vs Facility Biologic Infusion and ED or Hospital Admission on the Same or Next Day^a^

Characteristics	Infusions, No. (%)			aOR (95% CI)^b^	*P* value
	Total cohort	Home	Facility		
Infusions	752 150	34 078	718 072	NA	NA
Patients	57 220	3954	54 770	NA	NA
ED admission	2418 (0.3)	164 (0.5)	2254 (0.3)	1.63 (1.25-2.13)	<.001
Hospital admission	24 261 (3.2)	1336 (3.9)	22 925 (3.2)	1.21 (1.04-1.41)	.01
ED or hospital admission	26 544 (3.5)	1496 (4.4)	25 048 (3.5)	1.25 (1.09-1.44)	.002

Abbreviations: aOR, adjusted odds ratio; ED, emergency department.

^a^ ED and inpatient admissions include admission data for the same and next day after an infusion.

^b^ Odds ratio adjusted for age, sex, Charlson comorbidity score, year of infusion, disease specialty, frailty-related conditions, the top 7 most common infusion agents, and glucocorticoid use in the past 30 days.

### Secondary Outcomes

Home infusions were associated with 28% increased odds of discontinuation of the biologic after an ED or hospital admission (OR, 1.28; 95% CI, 1.08-1.51; *P* = .005) (eTable 3 in the Supplement), with 0.2 per 100 more individuals discontinuing after ED or hospital admissions for home infusions compared with facility infusions. There was no difference in the postinfusion mortality after infusions administered at home compared with a facility (eTable 3 in the Supplement).

### Subgroup Analysis

Patients who had an ED or hospital admission on the same or next day after biologic infusions had a higher Charlson comorbidity score compared with patients who did not have ED or hospital admission (mean [SD] score, 0.58 [1.13] vs 0.48 [0.94] for home infusions and 1.34 [1.62] vs 1.06 [1.31] for facility infusions) (eTable 4 in the Supplement). The risk of ED or hospital admission was higher with home infusions of tocilizumab (48 of 481 [10.0%] vs 1341 of 38 358 [3.5%]), vedolizumab (150 of 2681 [5.6%] vs 515 of 12 376 [4.2%]) and infliximab (1085 of 20 653 [5.3%] vs 13 191 of 373 985 [3.5%]) compared with facility infusions of these biologics, although the number of infusions of tocilizumab and vedolizumab was low (eFigure 3 in the Supplement).

The highest proportion of ED or hospital admissions occurred after the first infusion of a given biologic when administered at home, with 4.9% occurring after the first infusion (95% CI, 4.1%-5.7%), 4.3% after the second infusion (95% CI, 3.6%-5.2%), and 3.9% after the third infusion (95% CI, 3.1%-4.7%) of the same biologic (eTable 5 in the Supplement). For facility infusions, ED or hospital admission occurred after 3.7% of the first infusion (95% CI, 3.5%-3.8%), 3.7% of the second infusion (95% CI, 3.6%-3.9%), and 3.9% of the third infusion (95% CI, 3.7%-4.0%) of the same biologic.

## Discussion

To our knowledge, this is the largest study assessing the adverse events associated with infusion of biologics at home vs at a facility. In this study of patients with immune-mediated disease, infusion of biologics at home was associated with 25% increased odds of ED or hospital admission on the same or next day after the infusion compared with those given at a facility. We hypothesize that administration of biologics at home involves less intensive monitoring, less physician oversight, and lack of immediate access to urgent medical treatment in the event of an acute infusion reaction. This can result in delayed care and a more frequent need for escalation of care. Although permanent discontinuation of a biologic after ED or hospital admission was a rare outcome, it occurred more frequently after home infusions compared with facility infusions. Postinfusion mortality event rates were low, and there was no difference between home and facility infusions.

In our study, compared with patients receiving facility infusions, those receiving home infusions were younger and had fewer comorbidities. It is possible that patients receiving home infusions had less severe immune-mediated disease, as they received fewer prescriptions for glucocorticoids, although the data on glucocorticoids were not robust. Furthermore, although the patients receiving home infusions were younger and less comorbid, these patients were more likely to have an ED or hospital admission after biologic infusion in our study. This raises concerns about the possibility of increased usage of home infusions in older patients with more comorbidities and more severe immune-mediated disease.

Infusion reactions most commonly occur after the first infusion of a biologic.^15,23,24^ Additionally, patients with a prior infusion reaction are at greater risk of subsequent infusion reactions, and therefore less likely to be referred for home infusions.^15,23,24,25,26^ In our study, 25.5% of the patients in the home infusions group received their first infusion at a facility. This may have reduced the number of adverse events associated with home infusions in our study, and it is possible we may have underestimated the adverse events in the home infusions group. A retrospective study of 796 patients with inflammatory bowel disease who received infliximab at home reported rates of acute infusion reactions and ED visits similar to those previously reported for facility infusions.^27^ However, in this study, 86.8% of the home infusion patients received their first infusion at a facility, which likely biased the results in favor of safety of home infusion of biologics. These results highlight the need to carefully consider the place of service, particularly for the first infusion of a given biologic.

In our study, the risk of ED or hospital admission was noted to be highest after home infusions of tocilizumab, vedolizumab, and infliximab compared with facility infusions of these biologics, although the number of infusions of tocilizumab and vedolizumab was low. This finding is consistent with a small study of 138 patients with inflammatory bowel disease,^28^ which demonstrated that home infusions of either infliximab or vedolizumab were associated with significantly higher adverse outcomes compared with infusions administered at a facility.

This study has several strengths. First, we used a large claims database comprising patients from across the US over an 11-year time period. Most prior studies have been restricted to single institutions, in which local practice styles may influence the outcomes. Second, we included patients with a wide range of immune-mediated diseases. Other studies have focused on a single disease or group of rheumatological or gastroenterological diseases, even though the biologic agents used are similar across multiple specialties. By including a large number of immune-mediated diseases, our study had adequate power to detect meaningful differences in our outcomes. In an attempt to address the consequent heterogeneity in our cohort with this approach, we adjusted for the disease specialty and the top 7 most common biologics in the database. Finally, we were able to determine the proportion of first infusions that were delivered at home vs at a facility. Prior studies have been limited by a low number of first infusions delivered at home, which may have led to an overestimation of the safety of home infusions. In this large claims data study, a substantial proportion of the first infusions were administered at home, which may have contributed to the increased number of adverse events associated with home biologic infusions that was seen.

### Limitations

Our study has limitations. First, this is a retrospective study using claims data, and although we adjusted for age, sex, Charlson comorbidity score, year of treatment, disease specialty, Segal frailty index conditions, the top 7 most common infusion agents, and glucocorticoid use in the past 30 days, there may be residual or unmeasured confounders. Second, we did not adjust for background disease-modifying antirheumatic drugs such as methotrexate, although we are unaware of any data suggesting the usage of these drugs differs between patients receiving home vs facility infusions. Third, we did not have data on infusion premedications that could be associated with the rate of infusion reactions. However, we are unaware of any data suggesting that fewer premedications are given with home infusions compared with facility infusions. Fourth, we lacked data on history of prior infusion reactions. Given that patients who had prior infusion reactions would be more likely to receive subsequent infusions at a facility, we would expect this to only bias our results toward the null. Fifth, the infusion sequence data are limited by the fact that the first infusion observed in our data may not necessarily indicate the start of a new infusion if the patient recently transitioned to an Optum insurance plan. However, we would expect this phenomenon to be similar between patients receiving infusions at home or at a facility. Sixth, we did not have data on disease severity. Seventh, the primary indication for ED or hospital admissions that occurred on the same or next day after the infusion was not specified in the database. However, a similar trend was also seen for ED or hospital admission followed by permanent discontinuation of the biologic, suggesting an association with the biologic infusion.

## Conclusions

In this large retrospective cohort study, we found an increase in ED or hospital admissions after home infusion of biologics compared with facility infusion of biologics, even though the patients receiving home infusions were younger and had fewer comorbidities. Certain biologics may have higher adverse events compared with others, especially after the first or second infusion, and the decision to administer these biologics at home vs at a facility should be carefully considered. The proportion of infusions administered at home is projected to rise.^16,17,29^ This is in part owing to the COVID-19 pandemic and patient preference to avoid health care facilities and in part because of recent changes made by the Centers for Medicare & Medicaid Services expanding home care coverage.^18^ Rigorous assessment of the safety implications of administering biologic infusions at home is critical before they are expanded to older patients with more comorbidities and more severe immune-mediated disease.

## Notes

Supplement. **eTable 1.** Codes Used for Patient Inclusion **eTable 2.** Codes Used for Patient Exclusion **eTable 3.** Secondary Outcomes After Biologic Infusion Administration at Home Versus at a Facility **eTable 4.** Characteristics of Patients With and Without ED or Hospital Admission **eTable 5. **Infusion Sequence of a Given Biologic Administered at Home Versus at a Facility and the Association With ED or Hospital Admission **eFigure 1.** Flow Chart of Study Cohort Selection **eFigure 2.** The Percentage of First Infusions of a Biologic Delivered at Home Versus at a Facility **eFigure 3.** The Percentage of Infusions of a Given Biologic Associated With ED or Hospital Admission at Home Versus at a Facility
