# Supplementary material for: Comparison of Adverse Events Among Home- vs Facility-Administered Biologic Infusions, 2007-2017
Source: JAMA Netw Open. 2021 Jun 3;4(6):e2110268. doi: 10.1001/jamanetworkopen.2021.10268 (PMC8176330; doi:10.1001/jamanetworkopen.2021.10268)
Supplement: Supplement. — eTable 1. Codes Used for Patient Inclusion eTable 2. Codes Used for Patient Exclusion eTable 3. Secondary Outcomes After Biologic Infusion Administration at Home Versus at a Facility eTable 4. Characteristics of Patients With and Without ED or Hospital Admission eTable 5. Infusion Sequence of a Given Biologic Administered at Home Versus at a Facility and the Association With ED or Hospital Admission eFigure 1. Flow Chart of Study Cohort Selection eFigure 2. The Percentage of First Infusions of a Biologic Delivered at Home Versus at a Facility eFigure 3. The Percentage of Infusions of a Given Biologic Associated With ED or Hospital Admission at Home Versus at a Facility [file jamanetwopen-e2110268-s001.pdf]

Supplemental Online Content

Baker MC, Weng Y, Fairchild R, Ahuja N, Rohatgi N. Comparison of adverse events among home- vs facility-administered biologic infusions, 2007-2017. *JAMA Netw Open*. 2021;4(6):e2110268. doi:10.1001/jamanetworkopen.2021.10268

eTable 1. Codes Used for Patient Inclusion
eTable 2. Codes Used for Patient Exclusion
eTable 3. Secondary Outcomes After Biologic Infusion Administration at Home Versus at a Facility
eTable 4. Characteristics of Patients With and Without ED or Hospital Admission
eTable 5. Infusion Sequence of a Given Biologic Administered at Home Versus at a Facility and the Association With ED or Hospital Admission
eFigure 1. Flow Chart of Study Cohort Selection
eFigure 2. The Percentage of First Infusions of a Biologic Delivered at Home Versus at a Facility
eFigure 3. The Percentage of Infusions of a Given Biologic Associated With ED or Hospital Admission at Home Versus at a Facility

This supplemental material has been provided by the authors to give readers additional information about their work.

© 2021 Baker MC et al. *JAMA Network Open.*

eTable 1. Codes used for patient inclusion.

Biologic Infusions	
**J-Codes**	**Medication**
J9310	Rituximab
J2350	Ocrelizumab
J9301	Obinutuzumab
J1745	Infliximab
J3262	Tocilizumab
J0129	Abatacept
J1602	Golimumab
J0490	Belimumab
J2793	Rilonacept
J0638	Canakinumab
J3357	Ustekinumab
J2507	Pegloticase
J3380	Vedolizumab
J2323	Natalizumab
J3590	Unclassified biologic
**Rheumatology**	
**ICD-9**	**Diagnosis**
710.0	Systemic lupus erythematosus
714.0, 714.1, 714.2, 714.81	Rheumatoid arthritis
696.0	Psoriatic arthritis
710.2	Sjogren's syndrome
720.0, 720.1, 720.2, 720.81, 720.89, 720.9	Ankylosing spondylitis
714.30, 714.31, 714.32, 714.33, 714.81	Juvenile rheumatoid arthritis
714.89, 714.9, 714.90	Inflammatory polyarthropathy
710.3	Dermatomyositis
710.4	Polymyositis
135	Sarcoidosis
725	Polymyalgia rheumatica
446.5	Giant cell arteritis
446.4	Granulomatosis with polyangiitis
446.0	Polyarteritis nodosa
446.7	Takayasu's disease
136.1	Behcet's syndrome
446.21	Goodpasture's syndrome
447.6	Arteritis unspecified

© 2021 Baker MC et al. *JAMA Network Open.*

710.1, 701.0	Systemic sclerosis and circumscribed scleroderma
274.00, 274.01, 274.02, 274.03, 274.10, 274.11, 274.19, 274.81, 274.82, 274.89, 274.9	Gout
710.8, 710.9	Unspecified connective tissue disease
714.2	Adult-onset Still's disease
273.2	Cryoglobulinemia
279.8, 279.9	Disorders involving the immune mechanism, not elsewhere classified
**ICD-10**	**Diagnosis**
M32.1, M32.10, M32.14, M32.19, M32.8, M32.9	Systemic lupus erythematosus
M05.00, M05.09, M05.10, M05.19 M05.20, M05.29, M05.30, M05.39, M05.40, M05.49, M05.50, M05.59, M05.60, M05.69, M05.70, M05.74, M05.79, M05.80, M05.89, M05.9, M06.00, M06.08, M06.09, M06.20, M06.28, M06.29, M06.30, M06.39, M06.80, M06.88, M06.89, M06.9	Rheumatoid arthritis
L40.50, L40.51, L40.52, L40.53, L40.54, L40.59	Psoriatic arthritis
M35.00, M35.01, M35.02, M35.03, M35.04, M35.09	Sjogren's syndrome
M45.0, M45.1, M45.2, M45.3, M45.4, M45.5, M45.6, M45.7, M45.8, M45.9, M46.90	Ankylosing spondylitis
M08.09, M08.0, M08.20, M08.29	Juvenile rheumatoid arthritis
M06.4	Inflammatory polyarthropathy
M33.00, M33.01, M33.02, M33.03, M33.09, M33.10, M33.11, M33.12, M33.13, M33.19, M33.90, M33.91, M33.92, M33.93, M33.99, L94.4	Dermatomyositis
M33.20, M33.21, M33.22, M33.29	Polymyositis

© 2021 Baker MC et al. *JAMA Network Open.*

D86.9, D86.3, D86.0, D86.2, D86.1, D86.8, D86.89, D86.82, D86.86, D86.81, D86.84, D86.87, D86.85, D86.83	Sarcoidosis
M35.3, M31.5	Polymyalgia rheumatica
M31.5, M31.6	Giant cell arteritis
M31.30, M31.31	Granulomatosis with polyangiitis
M30.0	Polyarteritis nodosa
M31.4	Takayasu's disease
M35.2	Behcet's syndrome
M31.0	Goodpasture's syndrome
I77.6	Arteritis unspecified
M34.0, M34.1, M34.2, M34.81, M34.82, M34.83, M34.89, M34.9, L94.0, L94.1, L94.2, L94.3	Systemic sclerosis and circumscribed scleroderma
M10.xx, M1A.xx	Gout
M35.1, M35.8, M35.9, L94.8, L94.9	Unspecified connective tissue disease
M06.1	Adult-onset Still's disease
D89.1	Cryoglobulinemia
M30.3	Kawasaki disease
D89.9, D89.89	Disorders involving the immune mechanism, not elsewhere classified
**Neurology**	
**ICD-9**	**Diagnosis**
340, 341.0, 341.2, 341.9, 341.8	Multiple sclerosis
358	Myasthenia gravis
357.81	Chronic Inflammatory Demyelinating Polyneuropathy
356.9	Multifocal Motor Neuropathy
357.0	Guillain-Barré syndrome
**ICD-10**	**Diagnosis**
G35, G36, G37	Multiple sclerosis
G70	Myasthenia gravis
G61.81	Chronic Inflammatory Demyelinating Polyneuropathy
G60.9	Multifocal Motor Neuropathy
G61.0	Guillain-Barré syndrome
**Gastroenterology**	
**ICD-9**	**Diagnosis**
555.0, 555.1, 555.2, 555.9	Crohn's disease

© 2021 Baker MC et al. *JAMA Network Open.*

556.0, 556.1, 556.2, 556.3, 556.4, 556.5, 556.6, 556.8, 556.9	Ulcerative colitis
**ICD-10**	**Diagnosis**
K50.x	Crohn's disease
K51.x	Ulcerative colitis
**Dermatology**	
**ICD-9**	**Diagnosis**
696.1, 696.2, 696.3, 696.4, 696.5, 696.8	Psoriasis
694.0, 694.1, 694.2, 694.3, 694.4, 694.5, 694.60, 694.61, 694.8, 694.9	Pemphigus vulgaris and other bullous dermatoses
**ICD-10**	**Diagnosis**
L40.0, L40.1, L40.2, L40.3, L40.4, L40.8, L40.9	Psoriasis
L10.x, L11.x, L12.x, L13.x, L14	Pemphigus vulgaris and other bullous dermatoses
**Hematology/Oncology**	
**ICD-9**	**Diagnosis**
283.0	Autoimmune hemolytic anemia
287.31, 287.32, 287.49, 287.5	Immune thrombocytopenia
238.79	Castleman disease
**ICD-10**	**Diagnosis**
D59.0, D59.1	Autoimmune hemolytic anemia
D69.3, D69.41, D69.59, D69.6	Immune thrombocytopenia
D47.Z2	Castleman disease
**Nephrology**	
**ICD-9**	**Diagnosis**
583.1	Idiopathic membranous nephropathy
583.0, 583.1, 583.2, 583.4, 583.6, 583.7, 583.81, 583.89, 583.9	Nephritis
**ICD-10**	**Diagnosis**
N05.2, N04.2	Idiopathic membranous nephropathy
N00.x, N01.x, N03.x, N04.x, N05.x	Nephritis

ICD = international classification of diseases.

© 2021 Baker MC et al. *JAMA Network Open.*

**eTable 2.** Codes used for patient exclusion.	
**Hematology/Oncology**	
**ICD-9**	**Diagnosis**
200.2, 200.20, 200.21, 200.22, 200.23, 200.24, 200.25, 200.26, 200.27. 200.28	Burkitt lymphoma
200.5, 200.50, 200.51, 200.52, 200.53, 200.54, 200.55, 200.56, 200.57, 200.58	Primary central nervous system lymphoma
200.10, 200.11, 200.12, 200.13, 200.14, 200.15, 200.16, 200.17, 200.18	Lymphosarcoma
201.x	Hodgkin lymphoma
200.3, 200.30, 200.31, 200.32, 200.33, 200.34, 200.35, 200.36, 200.37, 200.38	Marginal zone lymphoma
200.4, 200.40, 200.41, 200.42, 200.43, 200.44, 200.45, 200.46, 200.47, 200.48	Mantle cell lymphoma
200.6, 200.60, 200.61, 200.62, 200.63, 200.64, 200.65, 200.66, 200.67, 200.68	Anaplastic large cell lymphoma
200.70, 200.71, 200.72, 200.73, 200.74, 200.75, 200.76, 200.77, 200.78	Large cell lymphoma
202.00, 202.01, 202.02, 202.03, 202.04, 202.05, 202.06, 202.07, 202.08, 202.80, 202.81, 202.82, 202.83, 202.84, 202.85, 202.86, 202.87, 202.88	Follicular lymphoma
200.4x, 200.8x, 200.7x, 200.1x, 200.3x, 200.5x	Non-follicular lymphoma
200.30, 203.80, 203.81, 273.3, 202.90, 202.91, 202.92, 202.93, 202.94,	Other and unspecified lymphoid malignancies

© 2021 Baker MC et al. *JAMA Network Open.*

202.95, 202.96, 202.97, 202.98	
204.1, 204.10, 204.11, 204.12	Chronic lymphocytic leukemia
204.90, 204.91, 204.92	Prolymphocytic leukemia of B cell type
204.00, 204.01, 204.02	Acute lymphocytic leukemia
204.20, 204.21, 204.22, 204.80, 204.81, 204.82, 204.90, 204.91, 204.92	Other lymphoid leukemia
207.3	Waldenström macroglobulinemia
273.1	Monoclonal gammopathy
203.00, 203.01, 203.02, 203.10, 203.11, 203.12, 203.80, 203.81, 203.82	Multiple myeloma
41.00, 41.01, 41.02, 41.03, 41.04, 41.05, 41.06, 41.07, 41.08, 41.09, V42.0, V42.1, V42.2, V42.3, V42.4, V42.5, V42.6, V42.7, V42.81, V42.82, V42.83, V42.84, V42.89, V42.9	Bone marrow or organ transplantation
279.5, 279.50, 279.51, 279.52, 279.53	Graft-versus-host disease
238.77	Posttransplant lymphoproliferative disorder
279.41	Autoimmune lymphoproliferative syndrome
446.6	Thrombotic thrombocytopenic purpura
**ICD-10**	**Diagnosis**
C83.7, C83.70, C83.71, C83.72, C83.73, C83.74, C83.75, C83.76, C83.77, C83.78, C83.79	Burkitt lymphoma
C83.3, C83.30, C83.31, C83.32, C83.33, C83.34, C83.35, C83.36, C83.37, C83.38, C83.39	Diffuse large B cell lymphoma
C83.5, C83.50, C83.51, C83.52, C83.53, C83.54, C83.55, C83.56, C83.57, C83.58, C83.59	Lymphoblastic lymphoma
C81.xx	Hodgkin lymphoma
C85.xx	Non-Hodgkin lymphoma
C88.4	Marginal zone lymphoma

© 2021 Baker MC et al. *JAMA Network Open.*

C83.1, C83.10, C83.11, C83.12, C83.13, C83.14, C83.15, C83.16, C83.17, C83.18, C83.19	Mantle cell lymphoma
C82.xx	Follicular lymphoma
C83.xx	Non-follicular lymphoma
C84.xx	Mature T/NK-cell lymphomas
C88.0, C88.2, C88.3, C88.4, C88.8, C88.9, C96.Z, C96.9	Other and unspecified lymphoid malignancies
C91.A, C91.A0, C91.A1, C91.A2	Mature B-cell leukemia Burkitt-type
C91.Z, C91.Z0, C91.Z1, C91.Z2	Other lymphoid leukemia
C91.9, C91.90, C91.91, C91.92	Lymphoid leukemia, unspecified
C91, C91.1, C91.10, C91.11, C91.12	Chronic lymphocytic leukemia
C91.3, C91.30, C91.31, C91.32	Prolymphocytic leukemia of B cell type
C91.0, C91.00, C91.01, C91.02	Acute lymphocytic leukemia
C88.0	Waldenström macroglobulinemia
D47.2	Monoclonal gammopathy
C90.00, C90.01, C90.02, C90.10, C90.11, C90.12, C90.20, C90.21, C90.22, C90.30, C90.31, C90.32	Multiple myeloma
Z94.0, Z94.1, Z94.2, Z94.3, Z94.4, Z94.5, Z94.6, Z94.7, Z94.81, Z94.82, Z94.83, Z94.84, Z94.89, Z94.9	Bone marrow or organ transplantation
D89.810, D89.811, D89.812, D89.813	Graft-versus-host disease
D47.Z1	Posttransplant lymphoproliferative disorder
D89.82	Autoimmune lymphoproliferative syndrome
M31.1	Thrombotic thrombocytopenic purpura

ICD = international classification of diseases.

© 2021 Baker MC et al. *JAMA Network Open.*

eTable 3. Secondary outcomes after biologic infusion administration at home versus at a facility.

	Total Cohort	Home Infusions	Facility Infusions	aOR	95% CI	p value
Number of infusions	752150	34078	718072			
Number of patients	57220	3954	54770			
ED admission + biologic d/c, n (%)	591 (0.1)	31 (0.1)	560 (0.1)	1.31	0.87-1.98	0.192
Hospital admission + biologic d/c, n (%)	2666 (0.4)	162 (0.5)	2504 (0.3)	1.26	1.05-1.51	0.013
ED or hospital admission + biologic d/c, n (%)	3222 (0.4)	192 (0.6)	3030 (0.4)	1.28	1.08-1.51	0.005
Post-infusion mortality, n (%)	309 (0.0)	11 (0.0)	298 (0.0)	1.51	0.57-4.01	0.40

ED and inpatient admissions include admission data for the same and next day after an infusion.
aOR = odds ratio adjusted for age, sex, Charlson comorbidity score, year of infusion, and disease specialty;
95% CI = 95% confidence interval; ED = emergency department; d/c = discontinuation.

© 2021 Baker MC et al. *JAMA Network Open.*

eTable 4. Characteristics of patients with and without ED or hospital admission.

	Home Infusion		Facility Infusion	
	**ED or Hospital Admission**	**No ED or Hospital Admission**	**ED or Hospital Admission**	**No ED or Hospital Admission**
Number of infusions	1496	32582	25048	693024
Percentage of infusions	4.4	95.6	3.5	96.5
Age, mean (SD)	42.5 (13.4)	43.2 (13.2)	52.7 (16.2)	51.3 (14.7)
Female sex, n (%)	858 (57.4)	19189 (58.9)	16997 (67.9)	475270 (68.6)
Charlson comorbidity score, mean (SD)	0.58 (1.13)	0.48 (0.94)	1.34 (1.62)	1.06 (1.31)

ED = emergency department; SD = standard deviation.

© 2021 Baker MC et al. *JAMA Network Open.*

eTable 5. Infusion sequence of a given biologic administered at home versus at a facility and the association with ED or hospital admission.

	Home Infusions Associated with Admission	Facility Infusions Associated with Admission
First infusion, % (95% CI)	4.9 (4.1-5.7)	3.7 (3.5-3.8)
Second infusion, % (95% CI)	4.3 (3.6-5.2)	3.7 (3.6-3.9)
Third infusion, % (95% CI)	3.9 (3.1-4.7)	3.9 (3.7-4.0)

95% CI = 95% confidence interval.

© 2021 Baker MC et al. *JAMA Network Open.*

eFigure 1. Flow chart of study cohort selection.

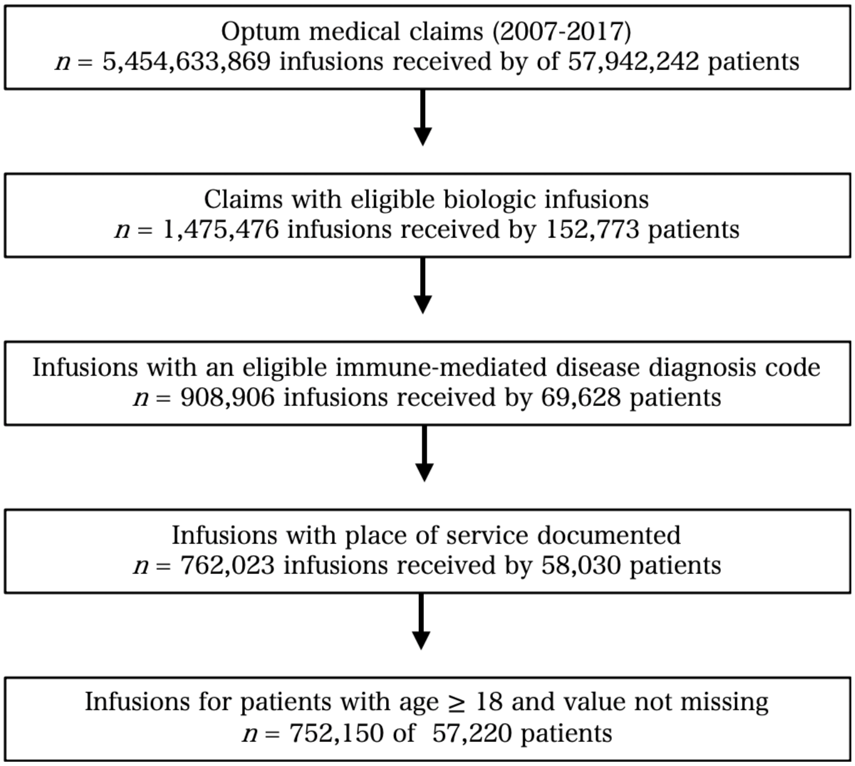

© 2021 Baker MC et al. *JAMA Network Open.*

eFigure 2. The percentage of first infusions of a biologic delivered at home versus at a facility.

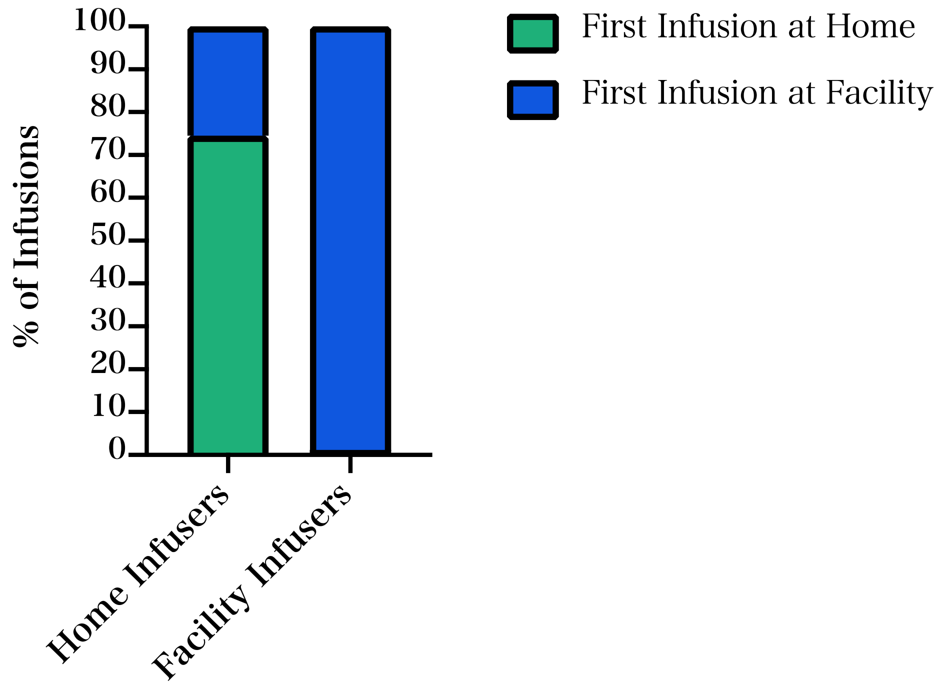

© 2021 Baker MC et al. *JAMA Network Open.*

eFigure 3. The percentage of infusions of a given biologic associated with ED or hospital admission at home versus at a facility.

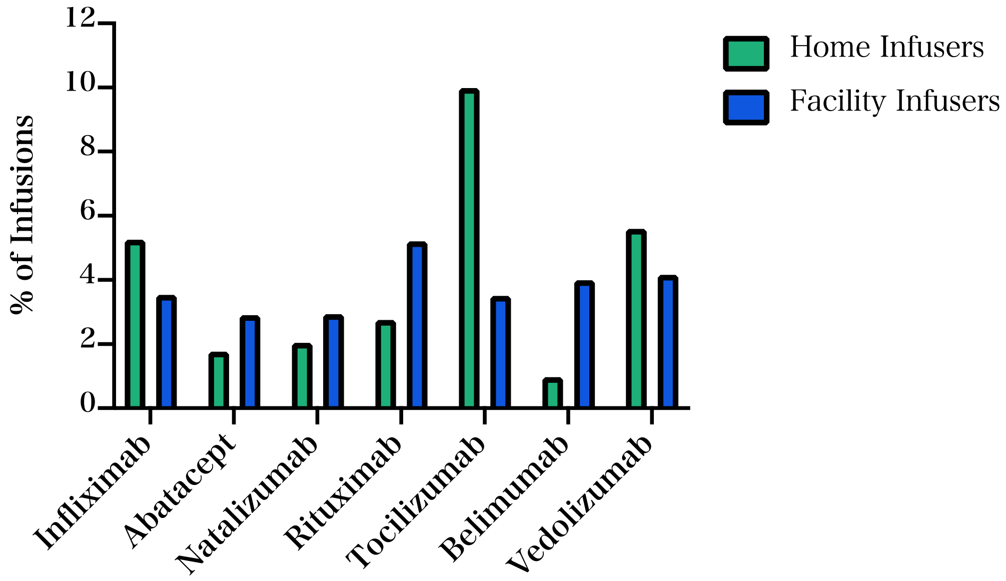

© 2021 Baker MC et al. *JAMA Network Open.*
